# Integrating Digital Assistive Technologies Into Care Processes: Mixed Methods Study

**DOI:** 10.2196/54083

**Published:** 2024-10-09

**Authors:** Sebastian Hofstetter, Max Zilezinski, Dominik Behr, Bernhard Kraft, Christian Buhtz, Denny Paulicke, Anja Wolf, Christina Klus, Dietrich Stoevesandt, Karsten Schwarz, Patrick Jahn

**Affiliations:** 1 AG Versorgungsforschung Pflege im Krankenhaus Departement of Internal Medicine University Medicine Halle (Saale) Halle (Saale) Germany; 2 Dorothea-Erxleben-Lernzentrum, Faculty of Medicine Martin-Luther-University Halle-Wittenberg Halle (Saale) Germany; 3 Institute for History and Ethics of Medicine Faculty of Medicine, Martin-Luther-University Halle-Wittenberg Halle (Saale) Germany

**Keywords:** digital assistive technologies, education concept, intention to use, learning effects, digital transformation

## Abstract

**Background:**

Current challenges in patient care have increased research on technology use in nursing and health care. Digital assistive technologies (DATs) are one option that can be incorporated into care processes. However, how the application of DATs should be introduced to nurses and care professionals must be clarified. No structured and effective education concepts for the patient-oriented integration of DATs in the nursing sector are currently available.

**Objective:**

This study aims to examine how a structured and guided integration and education concept, herein termed the sensitization, evaluative introduction, qualification, and implementation (SEQI) education concept, can support the integration of DATs into nursing practices.

**Methods:**

This study used an explanatory, sequential study design with a mixed methods approach. The SEQI intervention was run in 26 long-term care facilities oriented toward older adults in Germany after a 5-day training course in each. The participating care professionals were asked to test 1 of 6 DATs in real-world practice over 3 days. Surveys (n=112) were then administered that recorded the intention to use DATs at 3 measurement points, and guided qualitative interviews with care professionals (n=12) were conducted to evaluate the learning concepts and effects of the intervention.

**Results:**

As this was a pilot study, no sample size calculation was carried out, and *P* values were not reported. The participating care professionals were generally willing to integrate DATs—as an additional resource—into nursing processes even before the 4-stage SEQI intervention was presented. However, the intervention provided additional background knowledge and sensitized care professionals to the digital transformation, enabling them to evaluate how DATs fit in the health care sector, what qualifies these technologies for correct application, and what promotes their use. The care professionals expressed specific ideas and requirements for both technology-related education concepts and nursing DATs.

**Conclusions:**

Actively matching technical support, physical limitations, and patients’ needs is crucial when selecting DATs and integrating them into nursing processes. To this end, using a structured process such as SEQI that strengthens care professionals’ ability to integrate DATs can help improve the benefits of such technology in the health care setting. Practical, application-oriented learning can promote the long-term implementation of DATs.

## Introduction

### Background

Digital assistive technologies (DATs) offer novel possibilities for nursing and health care. Therefore, health care institutions must determine how to successfully implement digitization and adapt to the digital transformation in the health sector. Requirements include strategic planning, governing, organizing, controlling, orchestrating, and training technology-intense processes and services. Therefore, developing competencies among care professionals is necessary. Providing extra education on this topic will help care professionals appropriately implement and integrate DATs—as an additional resource—into their professional practices.

As no structured education concept for the implementation of DATs in nursing care currently exists, this study developed the sensitization, evaluative introduction, qualification, and implementation (SEQI) education concept. It then evaluated how the implementation of the 4-stage SEQI benefited nursing care by measuring the changes in care professionals’ intention to use DATs in long-term care facilities after its implementation. Long-term care facilities often fail to implement DATs because users have only a brief opportunity to familiarize themselves with and apply such technology. The population of interest for this study was defined as individuals trained and registered in a health or social profession who had worked in long-term inpatient facilities (care professionals). While the initial intention was to study only registered nurses as a target group, it quickly became apparent that this specification was an unrealistic inclusion criterion in current care practice. The care situation demonstrates that, in addition to nurses, other health care professionals, such as care assistants, nursing assistants, social workers, and physiotherapists, also provide a significant amount of care. It is evident that these individuals benefit equally from the implemented educational approach and, as a result, should not be excluded. In this study, a sample of care professionals from 26 long-term care facilities in Germany were examined using a mixed methods approach to understand changes in their intention to use DATs after the SEQI intervention. This study fills a gap in the literature by closely monitoring and evaluating the implementation of DATs using a structured approach.

Worldwide, health care systems are responding to the pressure created by increasing demand for care and the digital transformation of the health care sector [1,2]. Notably, DATs offer novel opportunities for promoting the independence and participation of older adults in long-term care [3] and improving their quality of life [4]. DATs can facilitate a range of activities associated with daily living, including smart medication management, digitally assisted fall prevention, and communication. DATs represent an evolution of assistive technologies that have been enhanced with digital capabilities [5]. Heinemann and Matusiewicz [6] pointed out that the digital transformation of health care and use of DATs can also be an opportunity to address the health care crisis. The term *health care crisis* is used to describe a phenomenon that has now affected many countries regardless of the structure of their social security systems, which all vary considerably yet are facing similar crises. These include an aging population, falling birth rates, increasing care needs, social isolation, declining social support networks, a shortage of nursing staff, and so on. The use of DATs is expected to benefit patients, care professionals, physicians, and health care organizations. Nursing care professionals are the leading user group among health care professionals; however, DATs can only be beneficial if care professionals accept and use them.

Some studies have discussed the use of DATs to support care professionals [7] in acquiring transformative competencies [8] and the relevant technological knowledge [9,10]. Care professionals’ expertise can reduce the risk of developing impractical and ill-suited technologies [2,11,12]. Thus, to realize DATs’ full potential, tailored solutions that address functional limitations are necessary, and DATs’ use should be preplanned and problem oriented. Research has established that systems intended for patient interactions, such as those that primarily support individuals in their activities of daily living (ADLs), should be the focus of DAT developments [5] as ADL assistance and support are frequent targets of nursing care. Therefore, it is important to take a differentiated look at the topic of robotics in nursing and, in addition to the technical shortcomings, examine other reasons why colleagues are not yet using robots in nursing care to the extent required. The question arises as to how DATs can be classified in the existing system of support for carers. This research focuses on systems to be used in direct patient interactions to support those affected, for example, in such ADLs as communication, self-feeding, and mobility. The targeted use for each application then has an assistive, supporting character and, therefore, is not new for care professionals.

According to the World Health Organization, *assistive technology* is an umbrella term that includes all adaptive and rehabilitative devices for supporting people with health impairments as well as their selection and use [13,14]. Assistive technologies have expanded to include a digital component that makes it possible to improve quality of life and opportunities for participation by promoting greater independence. Support always occurs when people can complete tasks and perform movements that would otherwise be difficult without technical support. At the same time, DATs reduce the need for formal health, support, and long-term care services by providing dependent care when self-care is no longer fully possible due to limitations.

An educated and trained use of DATs has the potential to reduce the high workload of nursing staff and other health care professionals.

Usability refers to the context-sensitive, application-oriented, and effective relationship between people and technology [15]. The lack of usability and implementation strategies for new technologies frequently results in unsuccessful implementation [16]. The most frequent causes of these failures are the lack of fundamental knowledge of the availability of DATs and poor understanding of their possible uses [9]. To date, innovative nursing technologies, such as those designed to prevent pressure ulcers and falls, provide supportive care for diabetes mellitus, address disorientation, and have predominantly been used as unconnected individual solutions on a person-by-person basis. To implement these technologies more broadly, the willingness of health care professionals to use them, as well as their actual use of such technology, must be analyzed. Providing opportunities to learn about the digitization of care approaches in the digital transformation era is also necessary.

Education on DATs that offers practical and actionable interpretations that help users better understand and use DATs in their daily practice should be provided to caregivers, care professionals, and health care professionals, which will ultimately lead to better care outcomes [17]. These interpretations are not directly applicable to caring practice. For practicality, arguments need to be transformed and excluded from their social scientific identity; they can then be reconstructed based on practice conditions in a way that is relevant and applicable to the practical situation. Doing so involves translating theoretical concepts and knowledge into practical actions that can be implemented in real-world situations. The transformation of theoretical knowledge into practical applications is an important component of any education approach aimed at integrating DATs into caregiving practices [17]. No theory-guided education concepts introducing DATs have been developed yet. Despite the identification of numerous challenges, strategies for the sound implementation of technology in nursing care can be formulated. These strategies should include training on digital skills [18], and the creation of a positive attitude among health professionals toward technology is essential. Nadav et al [19] argue that this can be achieved through the extensive introduction of professionals to technology operation. Albrecht et al [20] propose that this should be accompanied by a positive attitude toward technology. Therefore, understanding the factors influencing users’ intention to use DATs is key to ensuring the optimal integration of DATs within the health care system and achieving measurable benefits. A review of the German health care system (in which this study is also situated) reveals that surveys indicate a positive, open-minded, and inquisitive attitude toward new technologies among care professionals. For instance, one study demonstrated that care professionals tend to embrace DATs and perceive them as beneficial and user-friendly [21]. Conversely, respondents exhibited a more reserved attitude toward the use of robotics, with negative expectations of its use being more frequently expressed [21].

To address this issue, we propose a model based on the extended Technology Usage Inventory (TUI) to explore how users’ technology readiness and perceptions of DATs influence their intention to use them. This study’s results improved our understanding of caregivers and care professionals’ intention to use DATs and contributed to innovative research on the adoption of such technology. The proposed 4-stage SEQI is a novel education concept introduced as a form of learning. Its “evaluative introduction” and “implementation” stages are influenced by previous work [9]. *Evaluative introduction* refers to acquiring the competencies to assess whether DATs are suitable for addressing a functional nursing problem. *Implementation* is defined as on-site testing over a longer period in real use conditions. *Sensitization* means becoming aware of the digital transformation. *Qualification* is defined as the proficiency and expertise that individuals acquire to operate and use DATs in the given care context effectively and confidently. In summary, this mixed methods study examined whether a structured and guided education concept (ie, SEQI) can change care professionals’ intention to use DATs. The changes in intention to use and learning effects were captured through a quantitative survey of 112 care professionals. This study also evaluated the learning effects of the SEQI intervention based on guided interviews with 12 care professionals.

### Intention to Use DATs

In the health care context, previous studies have applied the technology acceptance model (TAM) and TAM 2 to explore acceptance and examine physicians’ [22-24] and nurses’ [25-29] intention to use DATs. Those studies have revealed nurses [30] and nursing care students’ intention to accept health care technologies [9,11,31,32]. Theories have conceptualized those factors (ie, barriers and facilitators) that influence the outcomes of implementation efforts spanning both generalized theory building and the development of practical approaches [33]. In particular, previous studies have found that user training and technology acceptance are key factors to successful implementation [33-35]. However, technical specifications and standards cannot simply be transferred to professional nursing from other fields [5]. The practical application of DATs or even the intention to use DATs is also significantly influenced by factors such as usability, usefulness, accessibility, and immersion; these factors are assessed using the TAM. Furthermore, psychological factors that affect the actual use of technology include acceptance, which is determined by personal attitudes such as curiosity, fear of technology, skepticism, and social norms [36], meaning that the individual characteristics of care professionals determine their intention to use DATs.

The acquisition of digital skills is essential for addressing the psychological factors that affect intention to use. The US health care system has slowly evolved from a system built on episodic and ambulatory in-person encounters to one that is digitally based, technology rich, and data informed [37]. The practical use of DATs requires an understanding of not only how to use them but also what opportunities and possibilities they offer for a customized and problem-oriented use in nursing care [37,38]. To achieve this, digital skills must be integrated more strongly into nursing curricula [2,12,39,40] and then expanded through advanced training and further education in later professional life. The nursing profession, for example, is facing a crisis in its education pipeline and professional development. Traditionally, formal health care education and the postgraduate novice-to-expert continuum have not emphasized technology and informatics as integral components of nursing [37]. Hence, solutions that enhance educational pathways should be explored, allowing care professionals to effectively practice in today’s health care environment under the digital transformation. It is helpful to distinguish the term *digitization* from the term *digital transformation*. *Digitization* describes a technical concept that includes software programs, corresponding hardware, and the translation of analog values into bits and bytes, whereas the term *digital transformation* generally describes changes that also relate to the values, attitudes, and mindsets of the professional groups concerned [41,42]. Targeted education programs should provide care professionals with the tools necessary for their profession, including the available DATs and their possible applications, as well as reflective competencies to evaluate and adapt these DATs for practical use in nursing. When properly used, DATs can provide opportunities for relief from providing care [29,33].

While implementing the SEQI education concept, researching and testing a possible education approach to introduce care professionals in long-term care to DATs was challenging. To develop a transformative implementation concept for DATs in the long-term care field, it was necessary to test care professionals’ understanding of DATs. The overarching project goal was to increase both care professionals’ intention to use DATs and, simultaneously, obtain their assessment of the practicality of the SEQI education concept. Currently available DATs are already an additional resource shaping the digital transformation of nursing processes. However, it was important for care professionals to understand the need to fit a specific DAT to a patient’s physical (functional) limitation or relevant care problem. Furthermore, initiating reflection and discussion among care professionals was crucial. As such, the following project goals were highlighted:

1. Focus on further education on DATs in real-world working conditions
2. Link theoretical and practical knowledge (theory-practice transfer)
3. Test currently available DATs
4. Focus on care professionals’ acquisition of knowledge and competencies
5. Build on care professionals’ expertise, knowledge, and learning habits
6. Improve practical testing experience to reduce uncertainty
7. Prepare for the digital transformation in the health care sector

### Theoretical Framework

Changes in professional, technical, and organizational conditions often lead to shifts in employees’ competencies or even require new competencies. The potential to sustainably change professional requirements, tasks, activities, and job profiles is a factor in the digital transformation of workplaces. In this situation, requirements for health care professionals’ competencies should also be evaluated. For example, the German Ethics Council calls for curricula to be supplemented to include new nursing techniques, including their ethical implications [12], to ensure the continuing education of care professionals. While transformative learning on how to manage the digital transformation of the health care sector is scarce, there is a great deal of interest in DATs as part of this transformation. Hence, the demand for professionals to acquire expertise in the field is increasing [9,43]. Thus, creating structured guidance to introduce care professionals to the use of DATs in nursing processes and planning is sensible. The theoretical considerations of transformative learning [44,45] provide a suitable framework for developing this type of guidance.

Beginning with the concept of lifelong learning [46], transformative learning allows earlier experiences to be reinterpreted and re-evaluated through the lens of experience-based assumptions and attitudes. Developing new perspectives through the dynamics of learning (intentional, intuitive learning) embedded in a problem-solving process is a focus of the concept [8,44,45,47]. This learning initiates a process of transformation, that is, the development of new perspectives on previously unquestionable attributions of meaning [8,47]. Care professionals can then embed the abstract construct of the digital transformation into their own experiences and reflect on it both as a starting point and as an end point for integrating technology into nursing processes [9]. By drawing on existing and experience-based knowledge, the possible applications for currently available DATs can be better assessed. The precise and solution-oriented use of currently available DATs as well as the development of new and innovative DATs offer great potential for defining nursing care problems both now and in the future.

### Problem Statement and Study Objectives

#### Overview

Despite the potential benefits of DATs in improving health care, their integration into nursing practices remains underdeveloped. The principal challenge is the absence of structured and efficacious educational methodologies that facilitate the patient-oriented integration of DATs in the nursing sector. The implementation of DATs is frequently impeded by the limited opportunities available to care professionals to become acquainted with and use these technologies effectively in actual clinical settings. This gap underscores the necessity for a comprehensive educational framework to guide the integration process.

#### Research Gap and Objectives

This study addressed the critical need for a structured approach to the education on and integration of DATs into nursing practices. The SEQI education concept was developed with the objective of providing a structured approach to support care professionals in understanding, adopting, and effectively using DATs. The objective of this study was 2-fold: first, to evaluate the impact of the SEQI education concept on care professionals’ intention to use DATs and, second, to assess the practical application and learning effects of this intervention in long-term care facilities.

#### Research Question

The primary research question guiding this study was as follows: how does the structured SEQI education concept affect care professionals’ intention to use DATs in long-term care settings?

#### A Priori Hypotheses

The first hypothesis was as follows: the implementation of the SEQI education concept is expected to significantly increase care professionals’ intention to use DATs.

The second hypothesis was as follows: care professionals who undergo the SEQI intervention will demonstrate enhanced understanding and practical application of DATs, leading to an improved theory-to-practice transfer of DATs.

This study aimed to provide a validated educational framework that can be widely adopted to facilitate the digital transformation in nursing and health care, ensuring that care professionals are well equipped to leverage DATs in their daily practices.

## Methods

### Overview

This mixed methods study used a combination of qualitative and quantitative data collection methods. The sequential explanatory design facilitates the determination of which quantitative results require further elucidation [48]. The sequential explanatory design with mixed methods comprises 2 distinct phases: a quantitative phase and a qualitative phase. The researcher initiates the study with a quantitative phase, which is followed by a second qualitative phase. The purpose of this second phase is to provide a more in-depth explanation of the initial results [48]. This research and development project ran from September 2019 to September 2022. It began with the question of what multimodal, transformative learning concepts presented in a structured manner could aid the digital transformation in the health care sector and encourage the use of DATs by health care professionals. It is recommended to understand multimodal educational offerings on DAT should present caregivers with a variety of approaches to addressing and thematically dealing with the topic. To ensure methodological quality, the Good Reporting of a Mixed Methods Study checklist [49] and the Mixed Methods Appraisal Tool [50] were used. The analysis of the in-depth interviews served to ascertain the needs and requirements of caregivers regarding an educational concept, thereby enabling the development of an intention to use DATs in the first place. This process of “connecting” the qualitative and quantitative phases was achieved through the sampling design. At the same time, the subject areas and subjects identified in the analysis of the interviews were used to estimate the dimensions and indicators of the results derived from the TUI questionnaire used in the quantitative part of the study. As noted previously, this study’s primary objective was to determine changes in the intention to use DATs. The secondary objective was to assess the learning effects following the principles of data triangulation [51,52].

### Survey Development

For the quantitative part of the study, a survey was conducted at 3 measurement points (T0, T1, and T2) at the end of stages 1, 2, and 4 of the SEQI education concept. The survey was developed using the TUI, a valid measurement instrument based on the TAM [36,53,54] that was created to evaluate the intention to use specific technologies by identifying factors that play a role in the technology adoption decision. Its basis is the theory of reasoned action, which holds that attitudes and behaviors are closely connected. Therefore, the behavioral intention to use a certain technology is determined by a person’s attitudes and social norms.

According to Kothgassner et al [36], the TUI distinguishes 3 main factors that affect technology acceptance and, thus, predict the actual use of technology: perceived usefulness, perceived ease of use, and attitude toward use. Perceived usefulness is defined as the subjectively perceived likelihood of improving performance by using a technology, whereas perceived ease of use is defined as the degree to which a product, system, or interface is designed and structured in a way that allows users to interact with it comfortably, intuitively, and with minimal effort. It encompasses elements such as user-friendly design, simplicity, and the overall accessibility of the system, contributing to a positive and efficient user experience. Both perceived usefulness and perceived ease of use directly influence an individual’s attitudes toward using a technology, which then directly affect their behavioral intention to use and, thus, their actual use of that technology [36].

The TUI consists of 30 items divided into 8 subscales. Of these subscales, 5 (curiosity, fear of technology, interest, immersion, and skepticism) have 4 items, whereas the accessibility, usability, and usefulness subscales each have 3 items. In this study, we adopted 7 of these subscales for our analysis: curiosity, fear of technology, interest, skepticism, accessibility, usability, and usefulness [36].

### Implementation of the SEQI: 5-Day Training Course

The 4-stage SEQI education concept was implemented in 26 long-term care facilities across the state of Saxony-Anhalt in Germany. For recruitment, a list of all long-term inpatient facilities in Saxony-Anhalt was made available by the discharge management of the University Hospital Halle (Saale). The list contained a total of 446 long-term inpatient facilities distributed across the state of Saxony-Anhalt. All facilities with a minimum occupancy of 50 beds (ie, 225/446, 50.4% of the facilities) were contacted and asked about their willingness to participate. In total, 26 facilities consented to participate in the study and were subsequently recruited. A total of 5 care professionals participated in each of the 26 training courses. On day 1 (sensitization), a workshop was used to sensitize and introduce participants to the digital transformation in health care and DATs. On day 2 (evaluative introduction), case vignettes and real-world nursing situations were discussed, and the care professionals selected a DAT based on the needs assessments of their patients. In addition, on day 2 (qualification), training was given on the proper use of the DATs. On days 3 to 5 (implementation), the selected DAT to be tested in the facilities was introduced.

This multistage structure of the SEQI education approach helped meet the core objective of this study, which was to raise awareness among care professionals of the digital transformation in health care and convey the use of DATs as a possible resource when planning nursing processes as this approach allowed for sufficient time for processing the extensive information provided in the 4 stages. Therefore, this training approach met care professionals’ desire for transparent information and training on new technologies.

**Figure 1:**
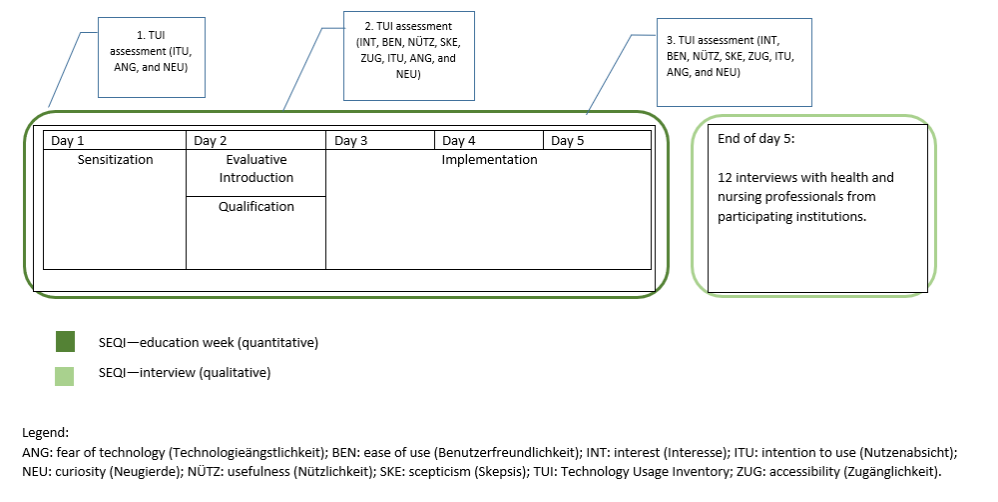
A schematic timeline of the sensitization, evaluative introduction, qualification, and implementation (SEQI) process.

In total, 2 researchers from the project team, a technician, and a nursing care researcher led the 5-day training course. Participants received additional informational material about the overall project and each DAT (see the next subsection). They were informed that they could contact the study team by telephone or email at any time during the trial period if they had any questions. Figure 1 shows the time frame of the SEQI intervention.

### Included DATs

The Assessment Instrument for Determining Care Dependency (BI) describes 8 modules [55-57]. In this study, these modules were understood as areas of care dependency within which specific nursing problems and a corresponding DAT assigned to achieve a defined nursing goal could be used [57]. On the basis of the assessment of the actual needs of the patients they were caring for, care professionals selected a suitable DAT. Six currently available DATs for use in long-term care facilities were selected:

1. A noninvasive sensor (DFree) that uses an app to determine bladder capacity and informs the user about the right time to go to the toilet [58]
2. A total of 2 robotic technologies belonging to the “social robotics” field (PARO and Pleo) [59,60]
3. A passive exoskeleton for relieving physical strain during demanding care tasks [61]
4. A mobile telepresence system with a self-balancing wheel and display for videoconferences [62,63]
5. Virtual reality applications for stress reduction and mindfulness [64]
6. A total of 2 communication robots for interaction using voice control and speech output (Pepper and Nao robots) [65]

### Assessment of the Change in the Intention to Use DATs

The TUI allows for inferences about the intention to use specific technologies [36], whereas other subscales assess the actual use of a technology (electronic supplement in Multimedia Appendix 1 and measurement time and instruments in Multimedia Appendix 2). The 3 measurements were taken at the beginning of the intervention (T0), the end of day 2 (T1), and the end of the intervention (T2). Thereafter, participants evaluated their experience with the DAT and the educational intervention. The data analyses were conducted by health and nursing scientists (MZ and CB) and a colleague from the technical team (DB).

### Assessment of the Learning Effects

A total of 12 guided interviews were conducted through theoretical sampling. The interviews were analyzed using systematic text condensation (STC) based on the work by Malterud [66,67]. The STC scheme, as proposed by Malterud [66,67], requires a structured approach to qualitative data analysis. This approach entails a bottom-up categorization process implemented inductively and, thus, hinges on a transparent and systematic methodology. This method begins with a comprehensive reading of the data to gain an overall impression followed by the identification of meaning units that relate to the research question. Subsequently, the meaning units are systematically coded into groups. These groups are then condensed, whereby the content is abstracted into a few comprehensive categories while the integrity of the original data is maintained. In conclusion, the essence of each category is synthesized into a theme or topic, thereby providing a clear and structured understanding of the data. This process guarantees that the analysis remains firmly anchored in the participants’ perspectives while enabling the formulation of meaningful interpretations.

### Guided Interviews

For the qualitative part of the study, 12 health care professionals participated in guided interviews. A total of 12 interviewees was deemed to be sufficient for facilitating an intensive data analysis, providing deeper insights, and achieving data saturation considering the resource constraints and research objectives. Interviewees were selected using convenience sampling. Statistical representativeness was not sought; rather, the interviews aimed to gather the specific knowledge necessary to understand participants’ assessment of the SEQI education concept.

The interviews were conducted after the SEQI intervention in 46% (12/26) of the participating long-term care facilities. In every second facility, the participating care professionals were asked whether one from the group would volunteer to be interviewed. The interviews were useful for obtaining both practically relevant background knowledge on the implementation of the SEQI education concept and guidance during the practical presentation of the research results [68]. The interviews focused on the meaningful, planned, and systematic integration of DATs into nursing processes from care professionals’ perspectives.

The interview guidelines were developed iteratively based on the SPSS method described by Helfferich [69]. The interviews were recorded and transcribed verbatim. The discussions were centered on the pivotal question of the suitability of the SEQI education approach for health care practice. The objective was to examine the concept in the context of the 5-day practical trial, identify potential improvements from a practical perspective, and enhance the content of the concept. The interviews were conducted by a health and nursing scientist (SH and BK) and a colleague from the technical team (DB and CK). The interviews were coded and documented independently by 3 members of the research team (SH, BK, and AW). The objective of data saturation was not initially established. The practical implementation of the saturation principle is contingent upon the availability of a somewhat larger sample and the flexibility to determine the number of interviews conducted. In this case, the sample size was not predetermined; rather, interviews were conducted until no new information or categories were added to the existing information or categories. In the pilot study, data saturation was not reached due to the project team’s awareness that data saturation is rarely achievable with regard to the comprehensive subject of the research. Instead, the interviews were to take into account the criterion of “internal representation,” which replaces the criterion of representativeness as a quality criterion for samples [69]. Given the inherent difficulties in achieving high concordance between coders, no effort was made to measure it. This is particularly the case in the first round of coding, which often leads to a revision of the category system. Therefore, the resulting data will depend heavily on the extent and differentiation of the category system developed. The data analysis followed the STC method determined by Malterud [66,67] using the MAXQDA analysis software (version 20.0.7; VERBI GmbH).

### Ethical Considerations

The ethics committee of the Medical Faculty of Martin Luther University Halle-Wittenberg approved this study on April 14, 2021 (approval 2021-021). The study was registered in the German Clinical Trials Register (DRKS00024967), and the protocol has not been published. Informed consent was obtained from all participants. No reward was given for participation.

## Results

### Survey Findings

A total of 122 participants were sampled from the 26 participating long-term care facilities. Of the 122 questionnaires returned, 10 (8.2%) were excluded from the analysis because they had incomplete responses or failed to select the specific DAT necessary for evaluating the intention to use DATs. In 41% (50/122) of the questionnaires, missing data were imputed using mean values (individual values were also attributed for various items). This procedure was performed after previous statistical consultation. As a simple random sample was used, the chosen imputation method was sufficient and did not affect the results. However, both simple and multiple imputations were performed to eliminate potential sources of error. The imputations were found to have only a minimal impact on the results. Half (62/112, 55.4%) of the participants were aged >41 years (Table 1). Most of the participants (78/112, 69.6%) had <20 years of professional experience.

**Table 1:** Sample characteristics (N=112).

Characteristic			Participants, n (%)
**Sex**			
	Female	87 (77.7)	
	Male	22 (19.6)	
	No answer	3 (2.7)	
**Qualification**			
	3-year duration of training	54 (48.2)	
	2-year duration of training	3 (2.7)	
	At least 1-year duration of training	6 (5.4)	
	Therapist (speech, occupational, or physiotherapy)	13 (11.6)	
	Social worker	17 (15.2)	
	Social or welfare worker	3 (2.7)	
	Other qualification	11 (9.8)	
	No answer	5 (4.5)	

### Intention to Use

Intention to use was scored using the selected 7 subscales of the TUI. The total score ranged from 0 to 300. High subscale scores indicated a high level of the corresponding construct, whereas low scores indicated a low level. The mean intention to use score was 232 (SD 55) out of 300 points at the beginning of the intervention (T0; Table 2) and remained at almost the same level throughout the intervention (T1 and T2).

No significance tests were conducted. Statements regarding significance were not necessary in this context because the trend was clear. Among the subscales, the participants were curious about DATs (21 out of 28 points), whereas their skepticism about DATs was low (13 out of 28 points; Table 3). In summary, the intention to use DATs and, thus, the predicted actual use of such technology in health care can be considered high.

**Table 2:** Results for the intention to use digital assistive technologies.

Time point	Scores, mean (SD)	Scores, median (IQR)
T0^a^	232 (55)	241 (203-275)
T1^b^	231 (66)	247 (208-280)
T2^c^	227 (72)	250 (187-288)

^a^T0: beginning of the intervention (n=111).

^b^T1: end of day 2 (n=112).

^c^T2: end of the intervention (n=112).

**Table 3:** Results for the intention to use digital assistive technologies by subscale.

	Score at T0^a^			Score at T1^b^			Score at T2^c^	
	Mean (SD)	Median (IQR)	Mean (SD)		Median (IQR)	Mean (SD)		Median (IQR)
Curiosity^d^	21 (5)	22 (18-24)	21 (5)		21 (18-25)	20 (5)		21 (17-24)
Fear of technology^d^	13 (6)	12 (7-17)	9 (4)		8 (6-12)	10 (5)		8 (6-12)
Interest^d^	—^e^	—	21 (5)		22 (17-25)	21 (5)		22 (18-25)
Ease of use^f^	—	—	16 (4)		17 (14-19)	17 (4)		17 (14-20)
Usability^d^	—	—	20 (5)		21 (17-24)	19 (6)		20 (15-24)
Skepticism^d^	—	—	9 (5)		9 (6-12)	10 (4)		9 (7-11)
Accessibility^f^	—	—	13 (4)		12 (10-15)	12 (4)		12 (9-14)

^a^T0: beginning of the intervention (n=111).

^b^T1: end of day 2 (n=112).

^c^T2: end of the intervention (n=112).

^d^Range 4 to 28.

^e^No measurement at time T0.

^f^Range 3 to 21.

### Interview Findings

As detailed in the Methods section, 12 health care professionals participated in guided interviews. The analysis of the interview data produced 14 codes that were condensed into 4 conceptual themes related to the learning effects: evaluation of the education concept (theme 1), effects on work and care structures (theme 2), need for reflection and discussion (theme 3), and improvement potential for health care and nursing care practice (theme 4). The 14 codes are presented in detail in Multimedia Appendix 3. Theme 1 describes the assessment of the SEQI education concept by health care professionals with a focus on the learning effects as SEQI focuses on training under real working conditions and, therefore, links theoretical and practical knowledge. Theme 2 addresses the impact of the education concept on work and care structures when using DATs. SEQI encourages the recapitulation and re-evaluation of work processes and care activities. The possibility to test already available DATs makes this recapitulation more realistic as it allows for the examination of integration possibilities under real working conditions. Theme 3 represents a synthesis of the preceding 2 themes. Care professionals assess application scenarios and determine the interactions among patients, DATs, and health care professionals based on the SEQI education concept. This entails a focus on knowledge and competence acquisition as well as the determination of interactions among patients. The opportunity to learn in a group of colleagues aligns with nursing learning habits and the availability of nursing expertise and previous knowledge. Theme 4 addresses the necessity to expand the scope of education and educate other professional groups on the subject matter. In this context, the dearth of DATs that are specifically tailored to patients’ functional limitations was also discussed. Practical testing experience reduces uncertainties and helps estimate which DATs can be used by other health care–related occupational groups. It is preparation for the possibilities of digitized health care, although it became evidently clear that the currently available DATs lack usefulness and usability for nursing care. This approach appears to be beneficial as it allows for the assessment of DATs using nursing expertise and the redesign of DATs to better align with the specific needs of nursing care.

## Discussion

### Principal Findings

The qualitative and quantitative findings reflect both the positive and negative aspects of DATs. To facilitate a more nuanced interpretation of the results, we will initially focus on the less complex quantitative survey data before subsequently turning our attention to the qualitative interview findings. This will facilitate the establishment of a clear link between the 2 sets of data. The survey results indicate that the reluctance of care professionals to implement DATs is not the primary cause of the issues that were identified. Rather, the perceived lack of usability and suitability was a significant contributing factor.

This mixed methods study was conducted to investigate the integration of DATs in nursing practices. This was achieved through the use of a structured education concept termed SEQI. Textbox 1 summarizes the key findings and insights derived from the study.

**Representative findings.**

- Willingness to integrate digital assistive technologies (DATs) in real-world working conditions. This study revealed that care professionals demonstrated a general willingness to integrate DATs into their nursing processes even before the implementation of the sensitization, evaluative introduction, qualification, and implementation (SEQI) intervention. This suggests that care professionals have a fundamental openness to adopting new technologies.
- Impact of the SEQI intervention. The SEQI intervention furnished care professionals with indispensable background knowledge and sensitized them to the digital transformation. The intervention enabled the participants to evaluate the suitability of DATs in health care settings more effectively, understand the qualifications required for their appropriate application, and identify factors that would facilitate their use and integration.
- Practical and application-oriented learning. This study underscored the significance of practical, application-oriented but structured learning in fostering the long-term integration of DATs. The SEQI approach proved effective in enhancing care professionals’ understanding and acceptance of DATs.
- Requirements for technology-related education built on care professionals’ expertise, knowledge, and learning habits. Care professionals learn to express specific ideas and requirements for technology-related care as well as education concepts. This feedback is crucial for developing effective training programs that address the practical needs and challenges faced by care professionals due to the digital transformation of health care.
- Active matching of support and needs to be prepared for the digital transformation in the health care sector. The active matching of support and needs is a crucial aspect of this process. This study highlighted the significance of aligning technical support with patients’ physical limitations (need to fit) and requirements when selecting and integrating DATs. This need-to-fit approach guarantees that the technologies used are beneficial and appropriate for the patients.

In the statistical methodology, the mean is a widely used measure of the central tendency of a given data set. However, the mean is susceptible to influence from values at the extreme high and low ends of the results. Consequently, the median is a superior measure of central tendency in instances in which a small number of outliers can significantly influence the mean. The median values identified in this study indicate a marginal increase in willingness to use, which is not apparent when interpreting the mean values. This indicates that the care professionals identified shortcomings in the selected DATs during the 3-day practical testing. A survey was conducted with 112 care professionals at 3 distinct measurement points to ascertain their intention to use DATs. The results indicated a positive shift in intention to use DATs following the implementation of the SEQI intervention. No sample size calculation or *P* values were provided. Nevertheless, the overall trend indicated an increase in acceptance, willingness, and intention to use DATs among the participants.

Qualitative interviews with 12 care professionals yielded further insights. The participants expressed appreciation for the SEQI program’s structured approach, particularly the evaluative introduction and on-site testing stages, which they found to foster confidence and competence in the use of DATs. In total, 4 primary themes were identified.

In theme 1, “Evaluation of the education concept,” the care professionals described the preparatory and theoretical introduction in stage 1 (sensitization), along with stages 2 to 4, as meaningful. Stages 1 and 2 (evaluative introduction) provided knowledge of the digital transformation and served as preparation for the practical experience in stages 3 (qualification) and 4 (implementation). The care professionals noted that a focused discussion of DATs is more effective than a pure knowledge transfer, such as only providing a user manual. Previous theoretical knowledge helps classify new information on DATs both ethically and normatively, and problems are related and supported based on the theoretical background as they arise, which is necessary for integrating DATs into practical work. Furthermore, the interviewees emphasized that practical teaching using case studies and group work is helpful for deepening understanding.

In theme 2, “Effects on work and care structures,” the care professionals reported that the use of DATs could enhance the quality of care and optimize work processes. However, it was also identified that the practical introduction of DATs presents challenges, such as the need for familiarity with new systems and the adaptation of processes.

In theme 3, “Need for reflection and discussion,” the care professionals indicated that the workshops facilitated reflection on the role of DATs in care facilities and on their own attitudes while using DATs. The opportunity to discuss these topics helped them gain a deeper understanding of the digital transformation and learn about different perspectives.

In theme 4, “Improvement potential for health care and nursing care practice,” the care professionals emphasized the importance of careful selection of DATs and the necessity of training on their proper implementation to improve the quality of care. In addition, cooperation between care professionals and technical experts was identified as a key factor in the successful introduction of DATs [40].

### Takeaways and Themes

The incorporation of structured educational methodologies such as the SEQI intervention is of paramount importance for the integration of DATs into nursing practices. The SEQI intervention provides care professionals with the requisite knowledge and skills to use these technologies in an effective manner. In addition, a positive attitude toward technology among health care professionals is essential for successful integration.

It is imperative to assess the usability of DATs in long-term care settings. The emphasis on practical application and real-world testing in SEQI proved to be a significant contributing factor to its success. By integrating theoretical knowledge and hands-on experience, SEQI ensures that technologies are tailored to meet specific patient needs, a recurring theme in our discussions. The structured and guided education concept of SEQI serves to enhance care professionals’ capacity to integrate DATs into their practices, facilitating this process through practical, application-oriented training. In the initial phase of the SEQI educational approach (sensitization), the theoretical underpinnings of digital transformation in health care were elucidated and deliberated with care professionals. Given that care professionals constitute the largest group in the health care system, they were encouraged to identify potential applications and areas of use for DATs based on their expertise (evaluative introduction). This process enabled them to evaluate DATs from the perspectives of their patients and the specific care challenges they face. The SEQI education concept can play an instrumental role of the profession as it invites care professionals to assess the usefulness of DATs. The structured theoretical knowledge transfer and the gradual transition from passive knowledge consumers to active users through consecutive training units were well received by the participants. This mutual approach proved conducive to the development of practical applications for DATs.

The qualitative findings provide information on 4 themes.

The SEQI initiative enhanced the comprehension of alternative courses of action and resolutions pertaining to the use of DATs in nursing procedures. The direct interaction during the testing phase enabled care professionals to assess the potential benefits and limitations of DATs, thereby fostering realistic and independent evaluations. The potential for technology to dehumanize care was countered through practical application testing, which demonstrated that care is fundamentally an interpersonal interaction that cannot be replaced by technology. Dehumanization is defined by Biniok [70] as a form of the denial of the human characteristics and qualities of other individuals. For example, social interactions and relationships could become devalued if care professionals were replaced by robots. The opportunity for direct testing highlighted the possibilities and shortcomings of DATs. The importance of the “human factor” for the high-quality provision of services became clear to the participating care professionals, and concerns about care professionals being replaced by DATs were refuted. The care professionals exhibited an awareness that care is fundamentally an interpersonal interaction that cannot be readily substituted by technology [12]. The interview data revealed that the care professionals’ lack of interest in DATs should not be interpreted as a general lack of interest [71-73]. This interpretation has arisen due to a methodological limitation in numerous previous studies in which care professionals were asked about a topic with which they lacked direct experience [29,74,75]. The lack of objective, tangible information on DATs and their potential applications makes it difficult for care professionals to formulate unbiased opinions. Consequently, they may only provide superficial responses to inquiries about their interest in DATs as it has been demonstrated that empirical evidence is essential for making well-informed decisions [76]. The focus was not on the DATs themselves but on the care professionals’ questions, conflicts, and behavioral uncertainties regarding DATs. This process-oriented approach to knowledge transfer, also known as the genetic method, aims not only to convey knowledge but also to acquire practical skills, thereby directing the focus to new or alternative situation interpretations.

The SEQI intervention can have a lasting impact when both cognitive transfer (knowledge, skills, and abilities) and emotional transfer (attitudes, values, motives, and feelings) are achieved [77]. An emotional transfer occurred when the relevance of DATs to specific care problems was perceived, thereby reducing skepticism and increasing willingness to engage with their implementation. SEQI helped defuse the often emotional discussion around robotics in health care because a realistic assessment of DATs was possible for the care professionals. One illustration of the emotional transfer achieved through practice with DATs was when a caregiver described the interaction of an introverted older adult with the PARO device. Upon being stimulated by the device, the older adult relinquished his self-imposed isolation, commenced laughing, and “began to tell stories.” Furthermore, a relatively young man with spinal cord injuries was able to experience a visit to a Rammstein concert through virtual reality immersion. The care professionals were impressed by the absence of any reservations or fears about the virtual environment.

Furthermore, SEQI facilitated the practical transformation of theoretical knowledge, as evidenced by the descriptions of necessary adjustments to workflows and care activities provided by the participating care professionals. Theme 2 (“Effects on work and care structures”) showed that the multiday on-site training was met with a positive evaluation, with participants noting the rarity of such opportunities for testing DATs in long-term care for older adults [78]. The results of the survey indicated that care professionals, the largest health care group, play a crucial role in the formation of sociotechnical care arrangements. The high scores on the interest and curiosity subscales and the low scores on the fear of technology subscale indicated that the participants were open to testing new technologies provided that they are usable and tailored to patient needs. In line with the theory of transformative learning by Mezirow [44], care professionals developed application ideas through reflective transformation, thereby ensuring long-term applicability in their professional practices. These considerations also extended to their own work processes, such as the integration of exoskeletons into shift planning or the logistics of outpatient care. To accomplish this, nursing professionals would be required to operate a vehicle while wearing the exoskeleton to visit and provide care for their patients and clients in their residences.

The survey results indicated the importance of actively involving nursing care professionals, the largest group of health professionals, in the design of socio-technical care arrangements (as evidenced by the high scores on the interest subscale and the low scores on the fear of technology subscale). In addition, the high scores on the curiosity subscale suggest that caregivers are open to trying new technologies, including DATs, if they are usable and tailored to the needs of target populations [79]. The consistently high scores on the overall intention to use scale were noteworthy as all participants demonstrated a willingness to use DATs at the outset of the study and retained this willingness even when the fit of the DAT to the patient was deemed to be inadequate. This indicates that care professionals perceive the potential for enhancing the suitability of DATs for their needs through participatory development and are willing to use them in the future.

In theme 3 (“Need for reflection and discussion”), the pragmatic aspects of the SEQI, such as sufficient time for questions, reflection during breaks, and mutual exchange with colleagues, were described in a positive light. It is imperative to reflect on DATs for the advancement of one’s professional capabilities, facilitating the expansion of personality traits and meta-competencies. The high score on the overall intention to use scale, despite the low fit of some DATs, indicates a fundamental interest in using technologies and a willingness to participate in their development and adaptation. This indicates that care professionals have a high level of self-responsibility and self-reflection about their professional practice and are willing to continuously educate themselves [80]. Through practical experiences and encounters, participants can easily acquire theoretical knowledge and apply it to their professional practice. If the acquired knowledge yields positive results when applied in practice, it is more likely to be remembered and applied in the long term. Therefore, practical training such as SEQI is often more effective than purely theoretical training as it offers the opportunity to directly implement learned knowledge and gain practical experience [80].

In theme 4 (“Improvement potential for health care and nursing care practice”), care professionals across the 26 facilities recommended an extended testing period on weekends to allow for a realistic assessment of DATs considering increased workloads and reduced staff capacity. Although facility management is ultimately responsible for procurement, care professionals acknowledged the necessity of balancing cost and benefit considerations. It was further proposed that additional health care professionals, such as nursing assistants, be trained in the use of DATs to ensure the maintenance of professional standards and appropriate delegation of caregiving tasks. Care professionals emphasized the importance of careful selection of DATs and the necessity of training on their proper implementation to improve the quality of care. In addition, cooperation between care professionals and technical experts was identified as a key factor in the successful introduction of DATs [40].

In conclusion, the SEQI educational concept effectively integrates structured education with practical application and real-world testing, thereby fostering positive attitudes toward DATs among care professionals. This comprehensive approach guarantees that DATs are adapted to the specific requirements of patients and residents and integrated into nursing practices, thereby improving the quality of care and optimizing work processes.

### Limitations and Strengths

The mixed methods approach, which focuses on qualitative, sequential exploration, was a strength of this study. The basic research design was critically reflected upon using the Good Reporting of a Mixed Methods Study checklist [49]. Furthermore, we classified and reflected on the quality criteria following Lincoln et al [81] and Lamnek [82]. The quality criteria of the survey were discussed using the Mixed Methods Appraisal Tool [50]. The openness of the mixed methods approach proved to be effective for this study considering the complexity and range of topics thus far underexplored. In particular, this approach allowed the researchers to provide a flexible response to the problems in the sampling strategy and the design of the empirical study.

This study had certain limitations. Primarily, it was conducted exclusively in long-term care facilities, and thus, the results are applicable only to this area. The recruitment of facilities was carried out exclusively at the management level, which may have introduced a positive selection bias (ie, only technology-friendly facilities were willing to participate). In addition, because all the visited facilities were informed about the observation element for ethical research reasons, the possibility of biased responses should also be considered. One of the key strengths of this study is that it marks the first time that caregivers have had access to DATs on a large scale and been able to test them in practice over a longer period in the context of nursing care. In preliminary work, the validity of the data would need to be critically questioned due to skepticism, which is particularly pronounced among care professionals in cases of lack of access to DATs or predefined media ignorance. However, research on DATs presents a complex picture. Although the self-image of care professionals indicates a high degree of conformity with traditional nursing practices [81], these practices have expanded, changed, and transformed the professional self-concept [33].

The question of whether it is reasonable to assume that nursing professionals are unable to manage intervention and interview situations independently is open to debate. This suggests that societal trends are employed to delineate uncertainties pertaining to the utilization of DAT in relation to the fundamental tenets of nursing practice, the incorporation of DAT into nursing processes, and the associated protection claims of individuals requiring care. In this context, societal trends are employed to describe nursing and healthcare professionals as occupying a subordinate position within a hierarchical healthcare system. This line of reasoning relies on a pervasive trope that portrays the purported uncertainty of nursing professionals with regard to the utilization of DAT. This gives rise to a debate about the possibility of technological reductionism undermining professional claims to individualized services and the expertise of nurses and healthcare professionals, particularly in the event that automated decisions are made by DATs in the future.

Moreover, there is a concern that the physical aspect of the interaction between care professionals and patients could be substituted by technology in the long term [83,84]. Within the group of participating care professionals, the fear of being replaced—a serious concern from the early days of discussion on DATs—is gradually giving way to the recognition of DATs as a useful complementary support tool [5,9]. Health care professionals are increasingly recognizing that digital technologies and robotics can complement and support their work rather than replace them. This indicates that the attitudes toward and perceptions of the role of technology in care have changed over time. Care professionals are increasingly seeing the interaction between technology and human care as improving caregiving for people [37]. This shift in attitudes suggests a growing recognition of the potential benefits of technology in nursing practices.

The primary challenge remains the development of methods to make DATs accessible and useful for care professionals in long-term care facilities. DATs have the potential to reduce the necessity for formal health, support, and long-term care services by assuming care tasks that are required when limitations prevent a patient from completing self-care independently. This, in turn, could result in a reduction in the workload of care professionals. Consequently, DATs are being developed in nursing to support patients and improve their compliance, which benefits care professionals. Thus, in conjunction with sufficient staffing, DATs can diminish the continuous work performed under time constraints and effectively digitize nursing care [5]. However, the impact of DATs on patients was not a primary focus of this study. Therefore, future studies should assess the extent to which the potential and effective alleviation of care professionals’ workload using DATs affects overall care quality.

Ultimately, the best DATs do not serve their purpose if they do not benefit the people being cared for. A strength of this study was that care professionals were granted broad access to DATs for the first time and, thus, experienced the actual usability of DATs in a real-world context. This indicates the conditions and situations in which care professionals accept or are skeptical about the use of DATs in long-term care facilities.

### Conclusions, Outlook, and Implications for Practice

This study revealed that care professionals are open to using DATs; however, they need information and knowledge on how to reflect on DATs critically. This study also highlights the effectiveness of the SEQI education concept for transferring theory into practice. Information, real-world practice, and learning are essential for reducing barriers and promoting an understanding of potential application fields and limitations. Our participants’ critical reflections revealed that currently available DATs only offer limited relief for care professionals as they merely support social care and daily life management. Hence, the targeted and patient-oriented use of DATs is necessary to promote critical reflection on the suitability of such technologies for nursing processes. The SEQI education concept can be used to strengthen care professionals’ competencies in dealing with DATs and enable a realistic assessment.

To achieve the long-term implementation of DATs in practice, practical and economic factors such as the creation and expansion of a comprehensive digital infrastructure must be considered. This study’s results underline the important role that care professionals play in the interprofessional team of health care professionals when using DATs as part of nursing processes. In this paper, the structured approach of SEQI was presented as a useful way to integrate DATs into nursing processes. The approach was positively received, and interest in DATs remained high throughout the study. The SEQI approach can help evaluate the suitability of DATs for identified functional nursing problems and integrate DATs into nursing processes. However, because of time restrictions for care professionals, it may be beneficial to involve other professional groups, such as social workers, in structured technology education. In addition, the exclusion of other health care professionals (eg, nursing assistants and station assistants) as potential users seems excessively limiting and should be avoided. In addition, while SEQI supports a more realistic understanding of DATs by care professionals, it can be used to stimulate discourse about DATs. As an education approach, SEQI has strong practical relevance, and its stages can be transferred to different nursing processes. This approach is easily implemented in long-term care facilities and could be included in the professional education of health care workers. Care professionals’ lack of experience with DATs highlights the importance of answering questions about the actual approaches regarding the sustainability of implementing these technologies in nursing practices. It is also essential to determine who qualifies to use these technologies.

The participants’ high willingness to use DATs in practice should be supported through holistic and application-oriented concepts that also consider ethical and normative aspects. SEQI, accompanied by the creation and expansion of a comprehensive digital infrastructure, can be implemented to build competencies to create the necessary conditions for the long-term implementation of DATs in practice. Regarding the use of DATs in nursing processes, care professionals play a central role among health care professionals.

## Appendix

Multimedia Appendix 1 Description of selected technologies tested during study period.

Multimedia Appendix 2 Supplement of measurement time during SEQI process.

Multimedia Appendix 3 Supplement Learning effects in detail.

## Abbreviations

ADL: activity of daily living
DAT: digital assistive technology
SEQI: sensitization, evaluative introduction, qualification, and implementation
STC: systematic text condensation
TAM: technology acceptance model
TUI: Technology Usage Inventory
