# Supplementary material for: Integrating Digital Assistive Technologies Into Care Processes: Mixed Methods Study
Source: JMIR Med Educ. 2024 Oct 9;10:e54083. doi: 10.2196/54083 (PMC11499723; doi:10.2196/54083)
Supplement: Multimedia Appendix 1 [file mededu_v10i1e54083_app1.docx]

*Online Supplement*

*Selected technologies*

1. **Dfree:** The DFree is a non-invasive sensor that is placed on the skin at the level of the bladder to measure the bladder fill level using ultrasound technology. Patients receive notifications via an app when the bladder reaches a certain level, allowing them to timely access a restroom. This DAT can support self-care competence.
2. **PARO & Pleo:** These technologies belong to the field of "social robots." They respond to touch and are designed to stimulate and engage individuals with cognitive impairments.
3. **Exoskeleton:** Passive exoskeletons are designed to provide back support. The Humic SoftExo is a wearable chest and back brace that adjusts to the body posture. The exoskeleton transfers the weight force exerted on the body's skeletal structure to the thigh supports, thereby redirecting it.
4. **Telepresence:** The "Double" is a telepresence system with a self-balancing wheel and a display for video conferencing. The device can be remotely controlled by the caller.
5. **Virtual Reality Application "VR Mindfulness":** The VR application is used in conjunction with a stand-alone VR headset. Virtual realities (VR) have been successfully used and further developed in the diagnosis and treatment of patients in the clinical field for the past 20 years [2]. The application and sensor used in the project are proprietary developments from the project.
6. **Communication Robotics (Pepper & NAO):** The primary function of the NAO and Pepper robots is communication and interaction through voice control and speech output. Additionally, nonverbal communication through body language is possible. To reduce negative reactions, the device has a gender-neutral appearance inspired by human features and the "baby schema".
