# Supplementary material for: Integrating Digital Assistive Technologies Into Care Processes: Mixed Methods Study
Source: JMIR Med Educ. 2024 Oct 9;10:e54083. doi: 10.2196/54083 (PMC11499723; doi:10.2196/54083)
Supplement: Multimedia Appendix 2 [file mededu_v10i1e54083_app2.docx]

*Online Supplement*

*Tabelle 4 measure times and instruments*

| Measure time | | TUI Version* | Skalen |
| --- | --- | --- | --- |
| T1 | Day 1 Workshop start before introducing the technologies | TUI I – Prä-Teil „original questionnaire (Prä/Post-Version)“ | NEU & ANG ITU |
| T2 | End Day 2 (Introduction of technologies) | TUI II – full „parallel-questionnaire (fullversion)“ | NEU & ANG INT, BEN, NÜT, SKE, ZUG, ITU |
| T3 | Day 3 to 5 End of the SEQI process | TUI II – full „parallel-questionnaire (fullversion)“ | NEU & ANG INT, BEN, NÜT, SKE, ZUG, ITU |
|  | * Kothgassner O, Felnhofer A, Hauk N. TUI technology usage inventory. Information- and Communication technology Applications: Research on User-oriented Solutinso. 2013. URL: <https://tinyurl.com/4vrtvrkj> [accessed 2024-04-29] | | |
