# Supplementary material for: Integrating Digital Assistive Technologies Into Care Processes: Mixed Methods Study
Source: JMIR Med Educ. 2024 Oct 9;10:e54083. doi: 10.2196/54083 (PMC11499723; doi:10.2196/54083)
Supplement: Multimedia Appendix 3 [file mededu_v10i1e54083_app3.docx]

*Online Supplement*

*Learning effects in detail*

1. Evaluation of the educational concept

The following presents the assessment of healthcare and nursing professionals regarding the four-step approach of the education concept. It refers to the structure of the entire week and evaluates the SEQI process as a whole. Since the value of a qualitative survey lies in the rich description of the experience, a small number of twelve participants are included in the survey. Generalizability of the results is not intended.

- 1. Gradual Development

The four-step structure is described as logically coherent and well-structured. The step-by-step approach breaks down the complex topic of digital transformation in healthcare into small sections. The individual parts, as well as their sequence within the training, help illustrate the topic and prepare for the implementation of Digital Assistive Technologies (DAT) in the care process, which is perceived as a new approach:

„Das war durchweg organisiert. Und so kennt man das im betreuten Wohnen nicht, ja [2021.08.20_10.22_01; Position: 25 – 26].“

„Ich fand es im Nachgang eigentlich wirklich VÖLLIG ausreichend und wirklich gut organisiert. Also: Erklärung, am Folgetag Einführung und dann Umsetzung (unv.) wunderbar [2021.08.20_10.22_01; Position: 162 - 162].“

However, it is noted in some cases that the first step of "raising awareness," where an initial theoretical overview of the topic was provided, is too extensive:

„Also für mich jetzt. Ich fand es, wie gesagt, also dann sehr kurz, sehr schnell, sehr viel so, diese ganzen Sachen dann an diesem Tag. Muss ICH sagen [2021.11.05_13.08_01; Position: 24 – 30].“

The temporal intervals and division of the individual process steps over several days are perceived as positive in order to allow space for reflection processes in the context of transformative learning:

„Den Prozess fand ich gut. Einfach, weil: Es ist schrittweise erfolgt. Man hatte auch Tage dazwischen, um noch mal selber nachzudenken und eigene Erkenntnisse aus der Verarbeitung zu ziehen [2021.09.10_14.23_01, Pos. 6].“

The division of the education approach into multiple days and four process steps is considered meaningful for workflow organization and planning purposes:

„Gerade der erste Tag ist ein guter Einstieg, weil er vergleichsweise kurz ist. Für uns ist das gut planbar. Am zweiten Tag wird alles gezeigt. Das ist auch noch mal halt super [ 2021.09.10_14.23_01, Pos. 6].“

The step of raising awareness and providing theoretical input on technology and knowledge transfer is characterized as important because it provides an initial understanding of the complex topics of digitalization and Digital Assistive Technologies (DAT).:

„Na erst mal dieses Ranführen, diese erste Hemmung abbauen [2021.08.06_12.53_01; Position: 42 – 43].“

„Also ich fand/ Es fanden eigentlich alle Mitarbeiter sehr gut. Es war auch diese/ Also am Anfang ja die Theorie, habe ich ja schon gesagt, war gut erklärt. Es war nicht zu viel, es war nicht zu wenig. Man musste jetzt nicht unbedingt vom Fach sein, na, ihr habt ja die Fachbegriffe erklärt [2022.03.18_14.19_01; Position: 12 - 12;].“

Furthermore, the intention of the professional group within the healthcare system to engage with aspects of digitalization is described as an added value:

„Der Aufbau von Ihnen am Montag war auch schön. Wie gesagt, da haben wir ja noch nicht viel gemacht. Wir haben uns kennengelernt. Es wurden die Sachen schon mal leicht vorgestellt. Warum es das geben könnte, WIE der Lauf der Dinge sein wird oder sein könnte: dass die Pflege wirklich – oder die Altenpflege speziell und Behindertenpflege – dann auch dolle mal nachziehen sollte wie die Ärzte und Krankenhäuser in der Technik. Ja, also das wäre wirklich schön, muss man ganz ehrlich sagen, in jeglicher Hinsicht [2021.07.16_10.55_01; Position: 54 – 54].“

The step of "evaluative introduction," which involves using an evaluation instrument such as the "New Assessment Assessment (NBA)" to assess the suitability of a technology for addressing a functional problem, is positively evaluated:

„Am zweiten Tag wird alles gezeigt. Das ist auch noch mal halt super. Auch, dass man sich GEMEINSAM Gedanken darüber macht: Was kann man wo vorher einsetzen? Also diese Evaluation im Beginnprozess [2021.09.10_14.23_01; Position: 6 – 6].“

The theoretical introduction is recognized as preparation for the practical part and is portrayed as meaningful:

„Da waren dann an dem Dienstag schon zwei, drei Bewohner mit dabei, als die Kollegen mit hier waren, die dann auch schon gleich das mit testen wollten. Und auch da war der Zugang/ Also es war seitens der theoretischen Vorbereitung Ihrerseits sehr gut [2021.09.03_11.48_01; Position: 29 – 31].“

The qualification step, which provides the first opportunity for active testing and familiarization with the technical devices, is an important component in the SEQI process:

„Ja. Sonst wären wir mit diesen Geräten (lachend) gar nicht klargekommen. Auch nur von so einer Anleitung glaube ich nicht, dass wir da richtig/ Ich denke, das ist wichtig. Weil Sie haben sie vorgestellt. Wir haben sie angefasst. Wir haben noch mal gefragt, auch mit der Brille, wie was genau das ist. Ich denke, ohne so was wird/ Wenn Sie die Geräte jetzt nur so hinstellen, nur mit der Bedienungsanleitungen, ich denke, da wird nicht so groß/ Werden wir nachgucken: „Oh, viel zu lesen.“ Ja. Nein [2021.10.22_11.06_01; Position: 55 – 56].“

The implementation step is seen as an opportunity for practical testing and a chance to question preconceived notions about digital and assistive technologies, as well as to reduce unfounded inhibitions.

„Also (auch?) definitiv ein Vorteil, weil es baut – ich sage mal – die verschiedenen Leute/ Also wir haben ja verschiedene Altersstufen, von den Mitarbeitern so wie von Bewohnern. Es baut einfach Hemmungen ab, ja? Also man hat so einen/ Viele haben vorgefertigt: „Ah Robotik Drei Punkt Null.“ Was weiß ich auch immer, was für Begriffe geprägt sind. Und es gibt Viele, die auch mit einem Abstand da so erst mal rangegangen sind, aber/ Also wenn man das umsetzen kann oder erproben kann, das hat halt wirklich bei Vielen diese Ängste abgebaut, denke ich. Oder diese, was man da so hat, Hemmungen [2021.08.06_12.53_01; Position: 30 - 30].“

1. Ethical and legal considerations:

Introducing an ethical perspective is seen as an important component for developing a nuanced understanding of potential application areas and user groups.

„Also was auch noch mal wichtig war, das war ja der ethische Aspekt dabei, ne? Es ist ja nicht alles für jeden bestimmt und man muss auch Distanz und Nähe wahren. Und man muss eben auch Entscheidungen treffen können als Pflegekraft: Ist das für denjenigen was, ne? [2022.04.01_13.10_01; Position: 23 – 23].“

Not only a cognitive transfer is addressed through practice, but an emotional transfer is also achieved. This helps to defuse the robotics debate by enabling a realistic estimation of Digital Assistive Technologies (DAT).:

„Ja, auf jeden Fall. Weil ansonsten wären wir, bin ich ehrlich, überfordert gewesen, wenn es jetzt einfach nur da gewesen wäre und/ Ja. Nein, war sehr gut geplant und organisiert“ [2021.08.20_10.22_01; Position: 18 – 18].

„Na erst mal dieses Ranführen, dieses erste Hemmung abzubauen“ [2021.08.06_12.53_01; Pos. 43 – 43]. (Sensibilisierung)

„Es hat mich eigentlich auch ein bisschen beruhigt, dass/ Ich habe erst gedacht: „Was kommt denn jetzt für ein Aufwand vielleicht auf uns zu?“ Ja? Irgendwelche Sachen noch ausarbeiten als Zuarbeit oder so was. Also da war ich dann eigentlich beruhigt. Denke: „Gut, okay. Wir dürfen dann selber tätig werden und wir müssen jetzt hier nicht noch irgendwelche theoretischen Sachen hier bearbeiten“ [2021.08.27_13.02_01; Position: 65 – 65].

„Aber man sieht halt auch die Grenzen des Ganzen. Also das ist uns, denke ich, im Umgang damit auch bewusst geworden: Es ERSETZT halt nichts, was wir jetzt im, ja, auf dieser emotionalen Ebene ganz anders mit den Leuten dann agieren. Das ist mal/ Das ist eine schöne Abwechslung, ja, was man sicherlich gut mit einbauen kann. Aber wie gesagt: in gewissen Grenzen. Also das kann keine Dauergeschichte sein. In keinem Fall. Das ersetzt den Menschen nicht“ [2021.11.05_13.08_01; Position: 45 – 45].

„Also (auch?) definitiv ein Vorteil, weil es baut – ich sage mal – die verschiedenen Leute/ Also wir haben ja verschiedene Altersstufen, von den Mitarbeitern so wie von Bewohnern. Es baut einfach Hemmungen ab, ja? Also man hat so einen/ Viele haben vorgefertigt: „Ah Robotik Drei Punkt Null.“ Was weiß ich auch immer, was für Begriffe geprägt sind. Und es gibt Viele, die auch mit einem Abstand da so erst mal rangegangen sind, aber/ Also wenn man das umsetzen kann oder erproben kann, das hat halt wirklich bei Vielen diese Ängste abgebaut, denke ich. Oder diese, was man da so hat, Hemmungen“ [2021.08.06_12.53_01; Position: 30 – 30].

1. Targeted knowledge and competency transfer

A targeted knowledge and competency transfer characterizes the training. However, the participants' assessment of the breadth of content in the theoretical introduction during the first step of raising awareness varies. On one hand, some participants desire a broader overview.:

"Aber ich denke schon: Die Möglichkeiten der Vielfältigkeit des Einsatzes war schon am ersten Tag sehr wichtig, dass man das (mitbekommt?), ne [2022.04.01_13.10_01; Position: 102 – 102]?“

On the other hand, it is suggested to limit the content of the theoretical introduction to the technologies that are specifically tested and explored in the training:

"Aber wenn ich fünf Sachen mitbringe, dann würde ich gerne von den fünf Sachen ganz viel intensiv hören, sodass ich die dann am nächsten Tag verwenden kann. Aber dann kamen wieder diese andere Information: „Wir hatten dann auch noch einen Wagen und wir waren auch in Halle.“ Und irgendwo ist dann auch meine, auf mich bezogen, ist dann mein Arbeitsspeicher auch irgendwann voll. Das/ Ich hoffe, Sie sehen (lachend) das jetzt nicht negativ [2022.04.01_13.10_01; Position: 100 – 100].“

Overall, it becomes evident that the participants form a heterogeneous group in terms of their prior knowledge:

"Genau. Also ich hatte ja den großen Vorteil, dass ich ein Stück weit die Technologie kannte. Schon einmal aus dem Besuch und einmal, weil ich persönlich sehr interessiert bin. Daher würde ich sagen, mein Lernerfolg bezieht sich hauptsächlich, mein persönlicher, auf die Anwendung der Technologien. Und der ist halt eigentlich sehr hoch, eben WEIL man sonst nie die Möglichkeit hat, so eine Technologie zu verwenden. Alleine schon auf Grund von Kostengründen. Ja, bei mir war es eher der praktische aufgrund des Vorwissens [2021.09.10_14.23_01; Position: 30 – 30].“

1. Impacts on work and care structures

The following describes the respondents' assessments regarding the impacts of Digital Assistive Technologies (DAT) on work and care structures.

- 1. Practical experience and integrative approach

The diverse practical experiences are considered crucial for learning how to handle Digital Assistive Technologies (DAT) competently:

"Und Vieles ist ja auch eigentlich selbst erlernen und erproben so ein bisschen [2021.08.06_12.53_01; Position: 41 – 41].“

"Für mich persönlich war von der Theorie mehr in die Praxis. Ich habe es jetzt gesehen, wirklich SELBER gesehen, wie das funktioniert und wie das ankommt [2021.10.22_11.06_01; Position: 48 – 48].“

In addition to involving staff members early on, the integration of clients and their relatives is crucial for openness in implementing the overall project. Considering the different perspectives of those affected by the use of DAT is made possible:

"Und natürlich war ganz wichtig, auch vorher, die Information, dass (man?) den Bewohner dabei hat, dass wir die Bewohner mit integriert haben, dass wir die Mitarbeiter schon im Vorfeld mit integriert haben, dass sie wussten überhaupt: Um was geht es? Die Erfahrungen haben wir ja jetzt erst in dieser Woche gelernt [2022.04.01_13.10_01; Position: 23 – 23].“

"Und was so schön war eben auch, dass viele Angehörige, die eigentlich nur – sage ich mal – nebenbei das gehört haben, erfahren haben, dass vielleicht was ist, weil der eine oder andere Angehörige das noch sagen konnte. DIESES Feedback der Angehörigen, das war auch so phänomenal, denn das sind ja eigentlich die Leute, die zukünftig VIELLEICHT in Pflegeeinrichtungen oder auf diese Assistenz-Hilfen angewiesen sind. Mit auf einmal im Boot waren und das auch so signalisiert haben, dass es was Tolles ist [2022.04.01_13.10_01; Position: 23 – 23].“

The integrative testing in practice helps to relativize expectations and overcome prejudices:

"Und sind dann aber bei der Ausprobierung oder – wie auch immer – Anwendung bei den Bewohnern auf einmal wirklich auf die/ Wir haben immer nur gedacht, die VR-Brille ist für UNS. Dass die Bewohner da aber so einen Spaß mit hatten und da auch mitgenommen worden sind. Da war der Pepper uninteressant, ja? Also das muss man ehrlich so sagen [2021.08.06_12.53_01; Position: 68 – 68].“

- 1. Complementary relationship

The practical testing of DAT made it clear to the participants that they have a complementary nature:

"Also das ist uns, denke ich, im Umgang damit auch bewusst geworden: Es ERSETZT halt nichts, was wir jetzt im, ja, auf dieser emotionalen Ebene ganz anders mit den Leuten dann agieren. Das ist mal/ Das ist eine schöne Abwechslung, ja, was man sicherlich gut mit einbauen kann. Aber wie gesagt: in gewissen Grenzen. Also das kann keine Dauergeschichte sein. In keinem Fall. Das ersetzt den Menschen nicht [2021.11.05_13.08_01; Position: 45 – 45].“

During the study week, there was an increase in interaction with clients:

"B2: Na weil wir im Normalfall gerade bei uns nach dem Frühstück ist ja/ GEHT JEDER SEINEN WEG. Von der Sache her wohnen die in ihren Wohnungen und wenn wir nicht nachm/ Nachmittags haben wir Beschäftigung. So haben wir sie jetzt nach dem Frühstück gleich hier behalten, haben sie aktiv mit beschäftigt. Ja. Also wir haben jetzt durch diese Studie ja auch natürlich diese Woche WESENTLICH mehr mit den Bewohnern gemacht [2021.08.20_10.22_01; Position: 48 – 48].“

- 1. Nursing expertise

The holistic perspective acquired through nursing expertise is described as an important support for the patient-centered use of DAT:

"Wir können das vielleicht anders einschätzen, weil wir noch die Diagnosen wissen und, und, und. Aber die Betreuungskräfte können das auch, weil die beschäftigen sich ja auch mit den Diagnosen, mit der Krankheit Demenz und, und, und, ja? Was/ Die haben ja da auch ihre Schulungen und so was. Das würde ich Ihnen bloß noch mal so mit auf den Weg (lachend) geben. Ja [2021.08.06_12.53_01; Position: 214 – 214].“

- 1. Technological competence and health promotion

In addition to the application-related competencies described above, the acquisition of technological competence is also mentioned:

"Also ich könnte mir vorstellen, dass es bei älteren Kolleginnen, zum Beispiel bei der Schwester xxx, auch ein bisschen in Sachen Technologiezuwachs geht. Also: Wie funktionieren diese Geräte? Was sind so die üblichen Sachen, um einen Fehler zu vermeiden? Ich meine, meistens ist es einfach, das Gerät neustarten. Ich kenne es schon, weil ich mich auch wieder ein bisschen mehr mit Technik beschäftige [2021.09.10_14.23_01; Position: 36 – 36].“

For specific devices, the directly experienced added value, such as in the field of prevention through the use of exoskeletons, is emphasized:

"Ja, auf jeden Fall. Also ich habe es ja selber gleich zum Anfang ausgetestet gehabt. Also zwei Tage am Stück. Es war auf jeden Fall eine Veränderung. Also man merkt es selber auch, dass man nach dem Tag auch anders geht. Ja? Also ich habe – mal ein banales Beispiel – auch noch ein paar Eier gepellt am Nachmittag. Das OHNE den. Da hat man dann schon mitgekriegt: „Ah, es macht Klick im Kopf. Man sollte sich doch wieder gerade hinstellen.“ Also man merkt es, auch dass psychisch da auch gleich eine Veränderung kommt, ja? Dass man gleich drüber nachdenkt: Man soll sich richtig hinstellen, besser hinstellen, kein Hohlkreuz machen. Ja, (unv) [2021.07.16_10.55_01; Position: 8 – 8]/“

1. Reflection and discussion needs

In this section, participants reflect on possible application scenarios and their suitability regarding the "care triad" (affected individuals, technology, healthcare professionals). The focus is on the possibilities and limitations of integrating DAT into care structures.

- 1. Usability and developement stage of technologies

It was emphasized that the usage of technology must align with the specific needs of the residents and individual challenges. The participants recognize the importance of tailoring the technology to the unique circumstances and requirements of each person:

„Also wir haben ja verschiedene Menschen hier bei uns. Manche sind sehr dement und haben das total abgelehnt. Aber manche, die halt im Bett liegen, die haben das sehr gut angenommen, obwohl sie es vielleicht nicht erkannt haben, dass es eine Robbe ist (unv.). Und, ja, unser (Hopspatient?) [Anm.: Patient mit hirnorganischem Pychosyndrom], von dem wir da geredet haben, dem haben wir wirklich auch Rammstein vorgespielt und er hat sich sichtlich gefreut darüber, über diese VR-Brille [2021.08.06_12.53_01; Position: 20 – 20].“

In addition to considering the health condition, it is important to take into account the general attitudes and overall interest of individuals. The participants emphasize the significance of considering personal preferences and ensuring that the technology aligns with the individual's values and interests:

"So, wie die Bewohner an sich auch TICKEN, wie ich die wahrnehme, wie die eingestellt sind, so haben sie sich auch zu der Technik verhalten. Also es gibt Menschen, die sind generell sehr tolerant. Auch, wenn sie schon älter sind. Die gibt es ja. Es gibt ja nicht nur die, die so Altersstarrsinn haben. Und wenn die so tolerant sind und interessiert sind, dann haben sie meistens diese Technik auch/ Waren sie neugierig und haben das nicht gleich abgelehnt. Das habe ich für mich so auf allen Bereichen eigentlich beobachtet [2021.08.27_13.02_01; Position: 4 – 4].“

- 1. Application areas for the use of technologies

Participants share their subjective experiences during the implementation phase of specific Digital Assistive Technologies (DAT). They reflect on the challenges, successes, and lessons learned while integrating and utilizing these technologies in their respective settings. These experiences provide valuable insights into the practical aspects of implementing DAT and shed light on the potential benefits and limitations of specific technologies:

„Der PARO ist super angekommen auf den Wohnbereichen. Gerade auch auf den Demenz-Wohnbereichen. Also die 3 ist ja so ein leichter Demenz-Bereich. Da war er super. Zum Beispiel unser Herr xxx, der weder lacht, noch redet, noch irgendeine Reaktion von sich gibt, den ganzen Tag nicht: dem haben sie den auf den Rollstuhl gesetzt und er hat gelacht. Und da habe ich/ Da hat/ Jetzt kriege ich schon wieder Gänsehaut. Und da habe ich Gänsehaut bekommen und habe gesagt: `Dafür ist so was da. GENAU für so was braucht man das´. Und da ist auch das Geld nicht zu schade, um auszugeben. Weil genau das sind die Momente, wo man ja an den Bewohner ran möchte. Und genauso war das auf der 5. Das ist Schwerstdemenz, also das ist wirklich/ Die erkennen sich jeden Tag neu. Die reden auch teilweise, also führen sie keine Gespräche. Dort ist wirklich viel Ruhe. Oder die spielen mit ihren eigenen Würfelspielen oder was auch immer. Und durch diesen Dino sind Gespräche entstanden. Und das war wunderschön zu beobachten. Das war ganz klasse. Und da sind auch die tollsten Fotos entstanden. Ich habe von diesem Wohnbereich noch nie solche tollen Fotos bekommen. Das war klasse [2021.09.17_11.27_01; Position: 4 – 4].“

- 1. Target audience of technologies

The presented and tested Digital Assistive Technologies (DAT) are considered meaningful. However, it is noted as a limitation that the majority of the DAT made available during the testing phase currently do not provide direct added value for indirect nursing care. This suggests that further development and refinement of the technologies may be necessary to fully leverage their potential in supporting nursing care:

„Aus dem Beschäftigungs-, Betreuungssektor MEHR als aus der Pflege. So, wie es jetzt aufgebaut ist. Also wenn, dann müsste da noch irgendwie/ Der Blasenkatheter ist ja – oder Blasensensor – ist ja EIN Punkt gewesen. Da müsste dann danach noch mehr kommen. Auch das Exoskelett ist ja zwar für beide nutzbar. Ich trenne das jetzt bewusst ein bisschen: Pflege und Beschäftigung. Aber es ist dann wieder/ In der Anwendung ist es dann eher beides wieder Richtung Betreuung [2021.09.03_11.48_01; Position: 76 – 76].“

It is emphasized that the currently available technologies primarily appeal to and are necessarily relevant for other professional groups involved in the care process. This highlights the importance of involving and educating these other professions to effectively utilize the technologies in the overall care context:

"B2: Und, ja, für das Weitere, wenn Sie die Projekte noch woanders machen, würde ich immer Ihnen auf den Weg mitgeben, dass Sie wirklich die Betreuungskräfte da mit reinnehmen.
B4: Das ist ein ganz wichtiger Faktor.
B2: Weil die sind wirklich diejenigen, die eigentlich mit diesen Materialien arbeiten. Weil die Fachkräfte haben dafür eigentlich keine Zeit [2021.08.06_12.53_01; Position: 212 – 214].“

- 1. Acceptance of residents and clients

Participants report a high level of openness and curiosity among residents towards digital and assistive technologies. The residents demonstrate a willingness to explore and engage with these technologies, which creates opportunities for meaningful adoption and integration into their daily lives. The positive attitude and curiosity of residents contribute to a more receptive environment for the implementation of DAT:

"Ja. War wirklich prima. Ist auch super bei den Bewohnern angekommen. Egal, mit was man oder was wir vorgestellt haben. Wir dachten ja wirklich so bei den Robotern, na dass da so ein bisschen die Berührungsangst war. Aber war nicht. Die fanden das toll. Ich bin mit den Dingern um die Ecke gekommen: „Oh, was ist denn das Und/ Ja." [2022.03.18_14.19_01; Position: 4 – 4]?“

Both the opportunities and limitations of using technology in nursing care are portrayed:

"Es gibt halt Bewohner, klar, die lehnen das ab. Die jetzt/ (War mir?) ganz klar. Welche, die sagen: „Nein, so ein Kinderkram.“ Und wie auch immer, na. Aber im Großen und Ganzen, denke ich, waren schon die Reaktionen positiv überwiegend. Aber man sieht halt auch die Grenzen des Ganzen [2021.11.05_13.08_01; Position: 45 – 45].“

1. Potential for emprovement in healthcare and nursing practices:
   1. Structures of procurement:

The cost-effectiveness ratio is critically evaluated by nursing professionals:

„Was ein bisschen abschreckt, das sage ich mal: der Preis und die Leistung, sage ich mal, ja? Also das passt noch nicht so überein [2021.07.16_10.55_01; Position: 28 - 28].“

The willingness to use Digital Assistive Technologies (DAT) as complementary resources is reflected upon in terms of the necessity for healthcare facilities to procure them.

„B2: „Es ist sicherlich auch ein finanzieller Aspekt, wa, dass man da in einem kleineren Pflegedienst sich dreimal überlegt, diverse Sachen anzuschaffen.

I1: „Das ist logisch. Der finanzielle Aspekt spielt bestimmt eine große Rolle.

B2: Na selbstverständlich. Weil natürlich die Pflege kann es nicht anwenden, wenn es die Einrichtungsleitung nicht anschafft [2021.08.20_10.22_01; Position: 189 – 193].“

„An sich, wenn das Interesse natürlich auch von der Leitung dann auch besteht so, was das angeht, ja, zur Erleichterung der Pflege, dann würde man sich natürlich auch daran setzen, ja, und mal gucken, was es da so gibt [2022.04.01_13.10_01; Position: 114 - 114].“

- 1. Involvement of other professional groups and the factor of time

The factor of "time" is addressed during the discussions. Currently available nursing assistants provide only indirect support for nursing care. The effort required to implement these assistants outweighs the benefits obtained. In terms of workflow organization, the use of Digital Assistive Technologies (DAT) primarily for communication purposes does not alleviate the workload for healthcare and nursing professionals. At the same time, participants describe the importance of testing the application of DAT in practice over an extended period to assess their effectiveness and impact throughout an entire shift, for example. This highlights the need for a longer-term evaluation to fully understand the benefits and challenges associated with implementing DAT in the context of time management and care delivery.

„Dafür war Aufwand und Nutzen dann einfach viel zu groß. So war es eine nette Abwechslung, aber zeitlich für UNS zu wenig, um das richtig zu planen, dass man das auch in der Nachtschicht mit nutzen könnte, dass die Kollegen da auch schichtübergreifend das mitbekommen könnten [2021.09.03_11.48_01; Position: 46 - 46].“

There is a desire to test the implementation of Digital Assistive Technologies (DAT) during periods of reduced staffing, such as weekends, when the workload tends to be more demanding.

„Und dadurch, dass es/ IN der Woche personell ist man ja auch besser aufgestellt als am Wochenende. So, dass man dann die Situationen auch in einem Umfeld nutzen kann, wo weniger Leute da sind, um sich auch um die Technologien zu kümmern und halt DIREKT in der Praxis mehr zu beurteilen [2021.09.10_14.23_01; Position: 6 – 6].“

- 1. Suggestions and comments on system optimization to improve suitability from the perspective of healthcare professionals

During the implementation phase, participants identify the potentials and weaknesses of Digital Assistive Technologies (DAT) through practical testing. Based on their experiences, they provide improvement suggestions to enhance the suitability of DAT for addressing relevant healthcare challenges.

„Das wäre vielleicht noch mal so ein kleiner Verbesserungsvorteil, dass man unten eine kleine Verstell-Schnelle macht, um die Größe noch mal einstellen zu können [2021.07.16_10.55_01; Position: 4 - 4].“

„Frag die doch mal, ob die jetzt eine Schildkröte gesehen haben oder ist da vielleicht ein Vogel? Und da hätte ICH es/ also aus meiner Perspektive schöner gefunden, wenn ich so ein kleines Tablet hätte, wo ich auch sehe, was DIE sehen. Dass (wir?) zum Beispiel dieses „Finde jetzt eine bestimmte Blume“, dass so ein bisschen Augentraining passiert

[2022.04.01_13.10_01; Position: 10 - 10].“

"Ja, also wir haben/ Also es gibt ja so was wie Weglauf-Matten und so was. Also die klingen dann, wenn derjenige da dann drüber geht. Und wir hatten uns eigentlich halt wirklich so gedacht jetzt, dass dieser Pepper – oder was es auch immer in dem Moment ist – die Leute anspricht, wenn die laufen, weglaufen und sagen: „Wo willst du hin? Kann ich helfen?“ Oder was weiß ich auch immer. Oder: „Kann ich dich mitnehmen?“ Natürlich kann er den nicht wirklich richtig an die Hand nehmen, aber irgendwie so ist halt das, was wir (mal?) gedacht hatten. Denn es würde uns ein bisschen den Alltag erleichtern, wenn wir im Zimmer sind und es ist keiner draußen auf dem Wohnbereich. Dass da einfach irgendeiner, wenn da jemand aufsteht, sagt: „Es kommt gleich jemand.“ Oder, oder, oder [2021.08.06_12.53_01; Position: 57 - 57].“
